# Proposing a Scientific and Technological Approach to the Summaries of Clinical Issues of Inpatient Elderly with Delirium: A Viewpoint

**DOI:** 10.3390/healthcare10081534

**Published:** 2022-08-13

**Authors:** Vincenza Frisardi, Matteo Nicolini, Nicola Cautero, Remo Ghirardelli, Federica Davolio, Mohamad Haouili, Mauro Barani

**Affiliations:** 1 Geriatric Unit, Neuro-Rehabilitation Department, AUSL-IRCCS Reggio Emilia, 46123 Reggio Emilia, RE, Italy; 2 Intensive Care Unit, Ospedale Civile di Baggiovara, 41125 Baggiovara, MO, Italy; 3 General and Emergency Surgery Department, Policlinico di Modena, 41125 Modena, MO, Italy; 4 Area Ig. Allev.e Prod. Zoot. Azienda USL Modena, 41125 Modena, MO, Italy; 5 Cooperativa Domus Assistenza, 41125 Modena, MO, Italy; 6 Unità Operativa di Radiologia, Ospedale di Sassuolo, 41049 Sassuolo, MO, Italy; 7 Information & Communications Technology (ICT), Policlinico S. Orsola-Malpighi, 40138 Bologna, BO, Italy

**Keywords:** delirium, disease management, patient care management, technology, healthcare, telemedicine

## Abstract

**Background/rationale:** Despite mounting evidence about delirium, this complex geriatric syndrome is still not well managed in clinical contexts. The aging population creates a very demanding area for innovation and technology in healthcare. For instance, an outline of an aging-friendly healthcare environment and clear guidance for technology-supported improvements for people at delirium risk are lacking. **Objective:** We aimed to foster debate about the importance of technical support in optimizing healthcare professional practice and improving the outcomes for inpatients’ at delirium risk. We focused on critical clinical points in the field of delirium worthy of being addressed by a multidisciplinary approach. **Methods:** Starting from a consensus workshop sponsored by the Management Perfectioning Course based at the Marco Biagi Foundation (Modena, Italy) about clinical issues related to delirium management still not addressed in our healthcare organizations, we developed a requirements’ analysis among the representatives of different disciplines and tried to formulate how technology could support the summaries of the clinical issues. We analyzed the national and international panorama by a PubMed consultation of articles with the following keywords in advanced research: “delirium”, “delirium management”, “technology in healthcare”, and “elderly population”. **Results:** Despite international recommendations, delirium remains underdiagnosed, underdetected, underreported, and mismanaged in the acute hospital, increasing healthcare costs, healthcare professionals’ job distress, and poor clinical outcomes. Discussion: Although all healthcare professionals recognize delirium as a severe and potentially preventable source of morbidity and mortality for hospitalized older people, it receives insufficient attention in resource allocation and multidisciplinary research. We synthesized how tech-based tools could offer potential solutions to the critical clinical points in delirium management.

## 1. Introduction

Delirium is a neuropsychiatric syndrome commonly observed in the hospitalized geriatric population [1]. The recent SARS-COV-2 pandemic demonstrated the presence of delirium in younger hospitalized patients [2]. Delirium can be a life-threatening condition with an increased risk of morbidity and mortality [3,4,5]. Preventing delirium is extremely important and a goal to be pursued in healthcare organizations. Multicomponent models of care exist to prevent the development of delirium in hospitalized patients [6] and manage patients with delirium at admission. Despite an enormous amount of literature on this topic, delirium remains a neglected area for intervention studies regarding how to properly allocate resources in general hospitals [7]. A French national survey on delirium screening and its management found a delirium underestimation despite the frequency of delirium as compared to the available data and how it was rarely screened with dedicated tools; furthermore, treatments reported often differed from the guidelines [8]. A prospective study in a British acute general hospital showed that only 27% of all older medical patients admitted to a hospital had assessment tool-diagnosed delirium [9], confirming how delirium is substantially underdetected. Uncertainties still exist on the optimal management of delirium. In 2013, an international European survey of practice exploring the attitudes of delirium recognition and management in healthcare providers with a special interest in delirium found areas lacking consensus (i.e., initial drug use, delirium workup evaluation, post-delirium follow-up), highlighting the gaps between clinical guidelines’ recommendations and clinical practice [10].

A road map of research priorities was proposed to advance the field in a systematic, interdisciplinary, and coordinated fashion [11]. Notwithstanding that, delirium continues to be mismanaged in everyday clinical settings and underestimated in healthcare planning. Starting from a consensus workshop sponsored by the Management Perfectioning Course based at the Marco Biagi Foundation (Modena, Italy) about clinical issues related to delirium management still not addressed in our healthcare organizations, we developed a requirements’ analysis among the representatives of different disciplines and tried to formulate how technology could support the summaries of the clinical issues. We analyzed the national and international panorama by a PubMed consultation of articles with the following keywords in advanced research: “delirium”,”delirium management”, “technology in healthcare”, and “elderly population”. This work aimed to summarize the critical points in delirium care. We tried to propose, in a multidisciplinary approach, how technology might help to overcome issues and challenges in this field. This paper is not a review but a synthesis of real clinical issues still affecting the healthcare professionals’ work and research field.

## 2. Definition, Clinical Presentation, and Epidemiology

Delirium is a severe disturbance in mental abilities that results in confused thinking and reduced awareness of the surrounding environment (loss of attention and alteration of the state of consciousness, orientation, memory, thought, perception, and behavior). An acute onset, with a transient and fluctuating course, characterizes delirium [12]. Usually, delirium develops within hours to days and represents a change from the patient’s baseline cognitive functioning. Most delirium presentations seem to be preceded by a prodromal phase with a change in “normal” behavior as in a state of unwarranted restlessness. Phenomenologically, delirium shows five features: cognitive deficits, attentional deficits, circadian rhythm dysregulation, emotional dysregulation, and psychomotor dysregulation. Phenotypically, there are at least five types of delirium. Based on its psychomotor characteristics, delirium could be (1) hyperactive, (2) hypoactive, or (3) mixed (a variable combination of both). Two other forms have been described: (4) the “catatonic variant” (an extreme form of hypoactive delirium) and (5) the “excited variant” (an extreme form of hyperactive delirium generally associated to drug abuse) [10]. There is also a sub-syndromal delirium, when delirium features do not meet all diagnosis criteria [13], and delirium superimposed dementia [14]. By definition, delirium is a transient syndrome. However, “chronic” or persistent delirium may be seen in a number of cases, such as those in people with baseline cognitive frailty or affected by acute cerebral injuries. The epidemiology of delirium varies considerably depending on the patient group’s characteristics, setting, diagnostic criteria, and detection tools [15]. The staff’s professional competencies to recognize delirium [16] and the efficiency in the reporting contribute to this variability [17]. Delirium point prevalence ranged from 9–32% (with the hypoactive subtype ranging from 23–78%) [18], 9–57% across hospital palliative care consultative services, and 6–74% in inpatient palliative care units. In palliative care, its prevalence was 42–88% [19]. These rates could be underestimated because many studies exclude patients with cognitive impairment or dementia who are particularly vulnerable to developing delirium [20]. An observational study assessing the prevalence of delirium among older patients living at home and periodically visited by their general practitioners (GPs) showed a high percentage (44.1%) among vulnerable and frail patients [21], while it was lower in other community settings (1–2%) [22].

## 3. Delirium Assessment and Reporting: From Research to Clinical Practice

More than 50 delirium assessment tools have been developed to screen and diagnose delirium [15]. A routinely pro-active assessment in high-risk patients or just in the case of suggestive symptoms of delirium is recommended. One of the major concerns in the clinical practice is the time it takes to fill out the assessment chart; ultra-brief instruments have been validated to overcome this issue [23,24]. Nevertheless, and in addition to clear NICE (National Institute for Clinical Excellence) guidelines’ recommendation [25], the frequency of delirium documentation in discharge summaries is variable, ranging from 7 to 44% [26], and only a small number of patients (3–16%) had a documented delirium diagnosis [27]. Omitted information in the discharge summary could affect patient safety [28]. The cumulative rate of delirium from the NHS (National Health System) demonstrated how it is still underreported [29,30]. The quality of discharge summaries varies across several specialties [31]. Compared to surgical services, medical services document delirium with more outstanding quality [27]; however, both services frequently lack the detail needed to ensure comprehensive follow-up [29,30]. Several factors might contribute to delirium underestimation: first, the lack of knowledge among health professionals of delirium (clinical and economic costs) and, secondly, the remuneration system based on the DRG/ROD (diagnosis-related group system, homogeneous clusters of diagnosis) is a classification system for discharged patients based on clusters homogeneous of diagnosis. Delirium is not an appropriate and profitable diagnosis in terms of reimbursement compared to other specific diagnoses [32], and inaccuracies in a discharge summary are deleterious for patients (hospital readmission and adverse events). However, Chuen and colleagues demonstrated higher rates of delirium documentation than in previous studies [17]. The discharge summary should be used to highlight necessary follow-ups by GPs or other providers in long-term care. The quality standards recommend communicating the presence of delirium to the patient’s GP [26]. Discharge summaries are a strategic time point for delirium management. Currently, the accuracy in reporting depends on several contributors such as the methodology and the time-consuming context in high-patient volume practices. Differences also rely on the quality of job organizations and the presence of trainees who complete discharge summaries. This may be optimized by introducing electronically distributed, standardized templates. Another crucial point is about the nomenclature and codification system. The Hospital Episode Statistics (HES), which are increasingly used in large-scale epidemiologic research, and ‘big data’ studies are based on the International Classification of Diseases 10^th^ Revision diagnostic code [33]. A comparison between research study estimates and routinely collected HES data for delirium found a 100–1000-fold disparity between the expected delirium rate (reported in prospective research studies) and the observed rates [34]. The lack of coding for delirium and other cognitive frailty syndromes will therefore lead to reduced hospital reimbursement payments, suboptimal case-mix adjustment, commissioning, and service planning, as well as the underestimation of the cognitive frailty burden in hospital cohorts [33]. Finally, there is a gray zone between “delirium” and “dementia with behavioral and psychological disorders”. These considerations shed light on the quality of the results from administrative data analyses. Multiple gaps remain, and improvements in this aspect are necessary to meet healthcare standards and give a basis for accuracy in database research.

## 4. Medium- and Long-Term Consequences of Delirium’s Mismanagement

### 4.1. Clinical Consequences

Patients with undetected delirium have a 6-month mortality rate, almost three times higher than those with detected delirium [35], and a hospital stay 2.3 times higher than those without delirium [36]. Further, an increased fall risk was observed in these patients [37]. Delirium exposes patients to a greater probability of being institutionalized [38], and it is a risk factor for dementia [39]. Preventing delirium reduces the cost of care in a short time but can also decrease the subsequent rate of dementia [40]. If we consider that no effective therapies for dementia are available, it will be necessary to intervene by strengthening prevention and reducing its burden. Decreasing the risk of developing cognitive decline by preventing delirium means that one-third of subjects destined for other factors to develop age-related dementia will probably delay the onset of invalidating symptoms, which would amount to a reduction in the incidence of dementia globally. It has been estimated that a 5-year delay in developing dementia is equivalent to halving the incidence of the disease [5].

### 4.2. Logistic and Economic Consequences

Some evidence showed the utility of a mobile, on-demand geriatric team to support other wards for inpatient elderly management. Despite the positive effects on clinical outcomes [41], one of the main limitations is the gap between recommended and concrete care. Just one consultation does not help clinicians to understand completely what triggered delirium. Furthermore, the reported adherence rates to geriatric suggestions are often suboptimal [41]. Sometimes noisy wards are not modifiable, representing a constant environmental trigger. Nevertheless, not all healthcare organizations have equipped delirium rooms or comfortable environments that reduce the risk of delirium incidence [42].

The high delirium prevalence in surgical populations [43] has stimulated debate to identify prediction rules for risk stratification and pre-habilitation programs aimed at preoperative optimization of a patient’s status. Many patients with successful immediate surgical outcomes subsequently succumb to delirium-related complications [falls, self-harm, infections, and pressure ulcers] and have considerable management challenges [44]. It follows that healthcare professionals’ job stress increases and job satisfaction decreases and, in turn, the quality of care provided decreases. Furthermore, the more stressful delirium management becomes, the more an ageist attitude spreads in the healthcare environment.

**Figure 1:**
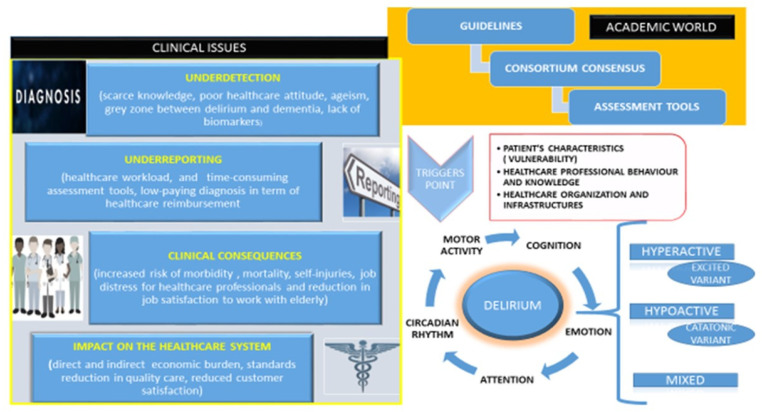
The wall of clinical issues about delirium. Phenomenologically, delirium shows five features: cognitive deficits, attentional deficits, circadian rhythm dysregulation, emotional dysregulation, and psychomotor dysregulation. Phenotypically, there are at least five types of delirium. Healthcare organizations may exacerbate the trigger point’s load with clinical issues that persist despite the increased academic knowledge on this hot topic.

Leslie et al. estimated hospital costs per day of hospitalization for patients with delirium [45]. Patients with delirium have higher unadjusted healthcare costs than patients without delirium (mean cost (SD), USD 146,358 (USD 140,469) vs. USD 94,609 (USD 80,648)). Healthcare costs increased directly and significantly with the delirium severity (none–mild, USD 83,534; moderate, USD 99,756; severe, USD 140,008) [46]. Furthermore, the healthcare costs attributable to delirium were estimated at USD 32.9 billion (95% CI, USD 25.7 billion–USD 42.2 billion) per year [46]. These evaluations should increase the “awareness” about delirium among providers, stakeholders, and healthcare policy makers to improve the efficiency of “allocation resources” and better care of hospitalized patients. Many healthcare organizations are reluctant to invest in technology that supports care despite the elderly representing an increasingly important share of the hospitalized population. In Figure 1, we report the synthetic framework around which this article was developed.

Providing comfort in the ICU setting generally means pain relief and end-of-life care; environmental factors are often neglected, despite the significant role of the environment on patients’ well-being and comfort [47]. By a multidisciplinary approach (clinicians, policy makers, healthcare managers, engineers, educational providers), patients admitted to the ICU may receive high-quality care while minimizing the profile for delirium onset.

## 5. Scientific and Technical Information Team (STIT) in Support to Delirium Management: From Diagnosis to Treatment

The development and organization of the health information systems have had a strong effect due to the emergence of renewal processes in the general health system. The informative computerized system allows the management of helpful information to simplify the healthcare processes; furthermore, it standardizes the communication of independent data [48]. Technological support in the health sector aims to pursue (1) continuity of care, (2) the centrality of the patient, (3) correct management of information flows, (4) optimization of the management processes of the various departments of the company, minimizing costs, (5) standardization of languages, data formats, and procedures; (6) optimization of communication, interoperability, and performance; (7) better management of guidelines, and (8) the promotion of medical research. Briefly, its role is to improve the health system’s quality [46]. In the field of delirium management, computer science is the harbinger of exceptional contributions not yet fully exploited. One of the innovative methods recently implemented during the COVID-19 pandemic is telemedicine. Telemedicine refers principally to the “integration, monitoring and management of patients, as well as the education of patients and health personnel, using systems that allow ready access to expert advice and patient information, regardless of where the patient resides” [49]. Starting from previous experience in other settings, there are many aspects of telemedicine that can be exploited for delirium management [50]. Since delirium often limits the return to one’s home environment, it is possible through telemedicine to support the caregiver with teleconsultation, monitor the patient, and provide for home hospitalization as a bridge between hospital and territory. The COVID-19 pandemic has allowed greater implementation of technological devices through video calls connecting family members and patients. Telemedicine offers numerous positive solutions in the delirium field and in the optimization of healthcare facilities in term of:

(1) **Prevention**: by the reduction in improper access to the healthcare services (teleconsultation), vulnerable people could avoid delirium triggered by the environment;
(2) **Cost reduction and optimization of resources**: to solve what it is solvable at home or in a nursing home by telediagnosis and telerehabilitation.

The lack of biomarkers or instrumental tests for early recognition of delirium also makes further management of the patient more difficult. In addition, clinical scales are time-consuming and health professionals may not appreciate the fluctuating nature of delirium. Circadian disorders are one of the core aspects of delirium. Therefore, implementing and applying biosensors (i.e., wristwatches) to monitor the sleep–wake cycle and impaired motor activity may help clinicians. Technology could be useful for collecting comprehensive information and contributing to research. Furthermore, it could serve to optimize resources (i.e., reporting patient changes that should lead to clinical surveillance to reduce the risk of clinical events such as falls, removal of venous access, etc.) [51].

This application finds its interest and value especially in the surgical departments, where the incident of delirium could affect post-operative recovery. The digital information system starts from patients’ data, and, by analyzing this information, it is possible to make decisions and perform an excellent person-centered action plan.

**Figure 2:**
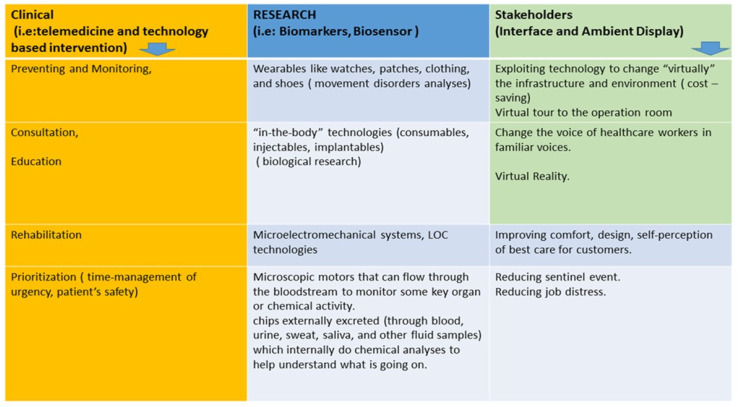
Technology advances could be useful to support clinicians in their activity thanks to the application of digital devices for telemedicine or tech-based intervention for monitoring, prevention, education, and clinical management of delirium. The importance of technology application comes from the lack of biomarkers for delirium diagnosis, and this determines the underestimation and undertreatment of this impacting syndrome. Technology sprouting will guarantee overcoming logistic obstacles to an aging-friendly environment realization in the healthcare setting, reducing the stress of healthcare workers when they deal with patients developing delirium.

We have synthesized, in Figure 2, the main pillars where scientific and technological advances could help to promote knowledge and management of care in the area of delirium.

## 6. Future Direction for a Better Clinical Management: The Intra-Hospital Delirium Pathway

The state of the art in delirium management is often not systematic, efficient, and effective enough. An external geriatric consultation occurs when the delirium has manifested and is fed by procedures of non-expert professional staff. The consultancy requested and carried out on a full-blown delirium managed by operators who do not belong to the geriatric team or are not adequately trained is only time and cost consuming. Currently, how delirium is managed favors underreporting as well as underdiagnosing. Hyperactive delirium is the leading cause of geriatric referral as it harbors discomfort for both the staff and patient. On the contrary, hypoactive delirium is not easily reported and no specialized examinations are required because it is not very disturbing. Additionally, an analysis of the consumption of goods and drugs is not deductible or frankly attributable to the delirium management; so, in the absence of a specific intra-hospital pathway, we will not have an appropriate economic estimate of delirium impact on our healthcare system. Consequently, without a measurable impact, we cannot plan further quality actions’ improvement and allocate the right resources. A structured pathway for inpatients with delirium should achieve the following goals (in the parentheses are possible ways to achieve them):

(1) Identification of at-risk (vulnerable) patients (detailed medial history, anti-cholinergic load assessment, evaluation of predisposing and precipitating factors);
(2) Carry out an early diagnosis of delirium, avoiding unnecessary diagnostic tests as a consequence of the accomplished, abovementioned point 1 (continuous training);
(3) The adequate treatment (de-prescribing, a careful pharmacological assessment, non-pharmacological approach) (education and training, healthcare professional attitude, resource allocation with staff qualitatively and quantitatively adequate);
(4) Comprehensive evaluation of patients admitted to the hospital and application of coping strategies for combative and agitated behaviors (delirium expert professionals, preferential care pathway for admitted elderly patients);
(5) High quality of documentation discharge about delirium (education, integration of digital system into clinical practice);
(6) Strengthen good practices to limit secondary injuries (self-harm) (avoiding restraints, inappropriate catheterization, nocturnal noise; having a respectful behavior; using ‘shock absorbent’ flooring in hospitals undergoing restructuring or simply floor mats with electronic sensors that activate when patient steps on it and by alerting the nurses station);
(7) Consolidate discharge planning to avoid institutionalization, re-hospitalizations, and worse outcomes (promoting robust networks with digital platforms among different healthcare professionals, social workers, patients’ associations, the public–private sector, and caregivers);
(8) Increase knowledge and empowerment of family and general practitioners (education, planning involvement actions);
(9) Provide an environment of care with technological innovations to monitor, make safe patient hospitalization stays, personalize care, and cure (involving and raising awareness among health policy makers, promoting pilot studies and international cooperation with industry);
(10) Follow-up of inpatients with delirium (digital tracking and strengthening the patient/caregiver–healthcare professionals’ alliance).

**Figure 3:**
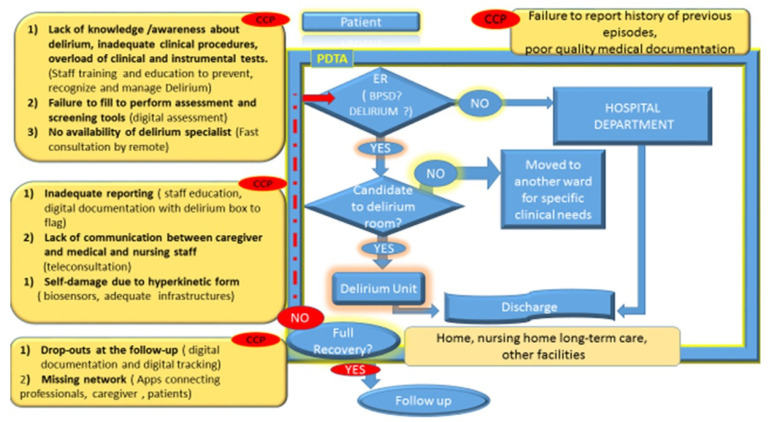
In a highly organized healthcare organization that is equipped with a delirium unit, within a well-codified PDTA (pathway for diagnosis, treatment, and assistance), delirium care management is characterized by several CCPs (critical clinical points). In the boxes, we report what the CCPs are and the criticism about what is the actual practice by suggesting possible solutions in the parentheses. At hospital admission, the lack of good documentation about the vulnerability of patients increases the risk to expose the elderly to the potential contributing factors precipitating delirium. Elderly patients with behavioral disorders are at risk of being diagnosed with dementia if personnel are not trained to recognize delirium. Sometimes, delirium occurs also in younger people, and they are managed in not-appropriate settings (delirium is mistaken for psychosis). Discharge is another critical point during the “hospital delirium journey”. In the end, people affected by delirium do have not a robust supporting network and they are lost at the follow-up. Therefore, the risk of cognitive decline is not promptly addressed.

Many threats exist in our health facilities that place older patients at high risk for delirium. In the meantime, the healthcare system does not achieve the highest quality of care. A room equipped for hyperactive delirium would be recommended. As we have highlighted the importance of the environment and training of all healthcare professionals, in accordance with desirable hospital aging-friendly practices, we proposed a potential flow chart within a PDTA (pathway for diagnosis, treatment, and assistance) to identify the critical moment for delirium mismanagement (Figure 3).

### Strenghts and Limitation

An international survey already showed barriers among clinicians to improving delirium detection. In particular, delirium awareness, knowledge/incompetence, lack of education, and lack of time for assessment were identified as the four main barriers. Previously, poor knowledge and education, staffing issues, and poor attitudes were the main barriers to improving delirium management [10]. Similarly, after discharge, only 6.6% of the participants reported a referral to a dedicated delirium follow-up clinic. Beyond highlighting these clinical issues unfortunately still present in our practice, our study shed light on the inconsistency of the classification system by claiming an update of the current reimbursement system. We call for a multidisciplinary action that involves stakeholders at different levels and healthcare managers to best allocate resources for delirium management. Infrastructures need to be adapted to avoid delirium triggers and healthcare staff needs to be educated for lower load deliriogenic behaviors. We are conscious that our study is only a viewpoint and delirium is a complex syndrome that needs to be addressed with a more comprehensive methodology. However, this is the first attempt to integrate the “4.0 dimension” in geriatric healthcare to solve outdated clinical issues and to provide key elements to support further education on this emerging topic.

## 7. Conclusions

Although all healthcare professionals recognize delirium as a severe and potentially preventable source of morbidity and mortality for hospitalized older people, it receives insufficient attention in resource allocation and multidisciplinary research. For this reason, stakeholders and policy makers need to address critical points from different perspectives. As delirium is a complex syndrome, a one-way solution is not conceivable.

Despite the efforts from scientific communities to provide guidelines for specific settings and situations, delirium is a clinical and organizational issue involving the entire healthcare system. Reducing clinical consequences for patients, professional staff, and the economic pressure on the healthcare system means reinforcing the professionals’ network by more robust discharge planning and a transition model care. Supporting vulnerable patients from admission to discharge means building a well-structured pathway (prevention, timely diagnosis, treatment, assistance, and follow-up measures) with a technological and digitalized contribution. Further research in this field is recommended including the impact on digital application to infrastructure and preventive strategies, digital recording to track delirium across the different facilities, and future changes of practice supporting vulnerable patients, their caregivers, and the healthcare professional staff.

## Data Availability

Not applicable.
